# *Euglena gracilis* Z and its carbohydrate storage substance relieve arthritis symptoms by modulating Th17 immunity

**DOI:** 10.1371/journal.pone.0191462

**Published:** 2018-02-01

**Authors:** Kengo Suzuki, Ayaka Nakashima, Masaharu Igarashi, Keita Saito, Makoto Konno, Noriyuki Yamazaki, Hiroaki Takimoto

**Affiliations:** 1 Euglena Co., Ltd., Shiba, Minato-ku, Tokyo, Japan; 2 New Drug Research Center, Inc., Toiso, Eniwa, Hokkaido, Japan; 3 Kitasato University, School of Science, Department of Biosciences, Kitasato, Minami-ku, Sagamihara, Kanagawa, Japan; University of South Florida St. Petersburg, UNITED STATES

## Abstract

*Euglena gracilis* Z is a microorganism classified as a microalga and is used as a food or nutritional supplement. Paramylon, the carbohydrate storage substance of *E*. *gracilis* Z, is reported to affect the immunological system. This study evaluated the symptom-relieving effects of *E*. *gracilis* Z and paramylon in rheumatoid arthritis in a collagen-induced arthritis mouse model. The efficacy of both substances was assessed based on clinical arthritis signs, as well as cytokine (interleukin [IL]-17, IL-6, and interferon [IFN]-γ) levels in lymphoid tissues. Additionally, the knee joints were harvested and histopathologically examined. The results showed that both substances reduced the transitional changes in the visual assessment score of arthritis symptoms compared with those in the control group, indicating their symptom-relieving effects on rheumatoid arthritis. Furthermore, *E*. *gracilis* Z and paramylon significantly reduced the secretion of the cytokines, IL-17, IL-6, and IFN-γ. The histopathological examination of the control group revealed edema, inflammation, cell hyperplasia, granulation tissue formation, fibrosis, and exudate in the synovial membrane, as well as pannus formation and articular cartilage destruction in the femoral trochlear groove. These changes were suppressed in both treatment groups. Particularly, the *E*. *gracilis* Z group showed no edema, inflammation, and fibrosis of the synovial membrane, or pannus formation and destruction of articular cartilage in the femoral trochlear groove. Furthermore, *E*. *gracilis* Z and paramylon exhibited symptom-relieving effects on rheumatoid arthritis and suppressed the secretion of cytokines IL-17, IL-6, and IFN-γ. These effects were likely mediated by the regulatory activities of *E*. *gracilis* Z and paramylon on Th17 immunity. In addition, the symptom-relieving effects of both substances were comparable, which suggests that paramylon is the active component of *Euglena gracilis* Z.

## Introduction

Rheumatoid arthritis is a disease that commences with inflammation of the synovial membrane located inside the joints and subsequently progresses to joint deformation due to the destruction of the bone and cartilage. The initial symptoms associated with the onset of rheumatoid arthritis are pain and swelling or stiffness of the finger and toe joints of the hands, feet, or both, symmetrically. Approximately 30% of patients exhibit an apparent improvement (remission) within 1 or 2 years after the onset, approximately 5–10% develop severe inflammation or symptoms shortly after the onset of signs, and other patients exhibit a repeated cycle of deterioration and remission with the progression of the joint deformation. Furthermore, approximately 1% of the population is believed to have recently experienced this condition [1,2], and an increase in the incidence is expected in future because of the aging population.

The disease etiology is not fully clear, but rheumatoid arthritis is considered an autoimmune disease caused by abnormalities of the immune system. Furthermore, it is known to complicate other autoimmune diseases including organ disorders, Sjögren’s syndrome, and Hashimoto disease. Recently, several antibodies have been used as drug therapies along with advances in the development of immunological investigations. The collagen-induced arthritis mouse model, which is known as a disease model that simulates autoimmune diseases and rheumatoid arthritis, has been used for the evaluation of medical products and health foods [3]. Collagens are proteins expressed in numerous body organs and tissues [4] that are classified depending on the chemical structures. Type-I collagen is located in the dermal layer, ligament, tendon, and bone while type-II collagen is mainly located in the articular cartilage.

A substantial number of antibodies against type-II collagen are known to exist in the serum, joints, and synovial fluid of patients with rheumatoid arthritis [5]. Moreover, it was reported that sensitization of rats and mice with type-II collagen, induced an arthritic condition resembling rheumatoid arthritis [5,6]. This evidence indicates that animal models established by sensitization with type-II collagen can be used as disease models of human rheumatoid arthritis for evaluating many medical products and health foods. *Euglena gracilis* Z is a microorganism classified as a micro-algae, and it possesses characteristics of plants and animals. It contains many nutrients including vitamins, minerals, amino acids, and fatty acids, and has shown efficacy in colorectal cancer [7] and type-II diabetes mellitus [8]. Furthermore, it has been reported as a food and nutritional supplement [9–13].

*E*. *gracilis* Z contains paramylon, which is a carbohydrate storing substance. Paramylon has a chemical structure of β-1,3-glucan and has been verified to exhibit potentiating activity [9], anti-atopic dermatitis effects [10], hepatoprotective effects on acute liver injury [11], anti-human immunodeficiency virus (HIV) activity [12], and antimicrobial activity [13].

In this present study, the symptom-relieving effects of *E*. *gracilis* Z and its carbohydrate storage substance, paramylon, on rheumatoid arthritis were evaluated in a collagen-induced arthritis mouse model. In addition, as a major constituent of *E*. *gracilis* Z, paramylon was further evaluated to determine its function and mechanisms of action.

## Materials and methods

### Test substance

The *E*. *gracilis* Z used in this study was a powder product, and the nutritional analysis results, which were similar to those of a previous report [8] are as follows: carbohydrates 29.4%, protein 42.3%, and lipid 19.0%. Approximately 70–80% of the carbohydrate content was paramylon, which was isolated from *E*. *gracilis* Z obtained from the euglena Co., Ltd., (Tokyo, Japan). The following usual method of preparation of paramylon was used. Cultured *E*. *gracilis* Z cells were collected by continuous centrifugation and washed with water. After suspending in water, the cells were broken down using ultrasonic waves and the contents, which contained paramylon was collected. To remove the lipid and protein, the crude paramylon preparation was treated with 1% sodium dodecyl sulfate (SDS) solution at 95°C for 1 h, and then at 50°C for 30 min with 0.1% SDS. After centrifugation, the paramylon was obtained, and further refined by repeated washing with water, acetone, and ether, sequentially.

### Reagents

To establish the collagen-induced arthritis model, type-II chicken collagen (C9310, Sigma-Aldrich), acetic acid (special grade, Wako Pure Chemical Industries, Ltd.), and Freund’s complete adjuvant (Difco) were used. For incubation of the lymphocytes, RPMI1640 medium (Wako Pure Chemical Industries, Ltd.), fetal bovine serum (Cell Culture Bioscience), and Gentamicin injection 40 μg/mL (MSD KK) were used. The cytokines were analyzed using the Bio-Plex Pro Mouse Cytokine Th17 Panel A 6-Plex Group I kit (Bio-Rad Laboratories).

### Animals

Male DBA/1J Jms Slc (specific pathogen-free, 6 weeks old) mice were purchased from Japan SLC Inc. The animals were housed in a breeding cage (CLEA Japan, Inc.) in groups in an animal room maintained at a temperature of 24 ± 2°C, relative humidity of 50 ± 5%, full ventilation, and illumination at 6:00–20:00. The animals were fed a radiation-sterilized powder diet (CE-2, CLEA Japan, Inc.) and were provided with tap water *ad libitum* for drinking water.

The individual animals were identified using the ear-punch method. The day of receipt was designated as day 0, and the animals were quarantined until day 7. Their general condition was monitored daily, and all animal experiments were conducted with the approval of the Animal Ethical Committee of Kitasato University.

### Induction of collagen-induced arthritis

The collagen-induced arthritis model was established in animals in the experimental groups: Control, *E*. *gracilis* Z (E), and paramylon (P) groups. Type-II chicken collagen was dissolved in 0.01 mol/L acetic acid aqueous solution to a concentration of 2 mg/mL. To this solution, an equal amount of Freund’s complete adjuvant was added to prepare the emulsion (collagen: 1 mg/mL). This emulsion was injected subcutaneously into the base of the mouse tails at a dosing volume of 0.1 mL/mouse (0.1 mg as collagen) using a 26G injection needle and a locking glass syringe under anesthesia with isoflurane. Three weeks later, the same dose was repeated (booster immunization). The animals in the Normal group were not sensitized to induce arthritis.

### Administration of test substance

Each test substance was mixed in the powdered diet, CE-2, at a concentration of 2% and the dietary administration to Groups E and P was initiated 5 days after the booster immunization. The Normal and Control groups were fed the intact powder diet, CE-2, without any supplementation.

### Body weight measurement

The body weight was measured once a week from the end of the quarantine period until the boost immunization day, and then three times a week after the booster immunization.

### Observation of symptoms of arthritis

After the booster immunization, the condition of the extremities associated with arthritis were evaluated three times per week (Monday, Wednesday, and Friday) according to the scoring criteria of Bernadeta et al. [14], and the total score of all four extremities was calculated using the following scale as follows: 0: no evidence of erythema and swelling, 1: slight redness/swelling of one small joint such as phalangeal joint, 2: redness/swelling of two or more small joints, or a large joint, 3: redness/swelling of one extremity, and 4: maximum redness/swelling of the whole region of one extremity.

### Analysis of serum antibody titer and cytokines

After termination of the test substance administration, laparotomies were performed under inhalation anesthesia with isoflurane, and blood samples were collected from the abdominal vena cava. Then, the animals were euthanized by exsanguination, and then the inguinal lymph nodes and knee joints were harvested. The serum was separated from the blood samples by centrifugation to quantify the IgG levels using an enzyme-linked immunosorbent assay (ELISA).

The lymphoid cells were separated from the inguinal lymph nodes and then divided into three portions; each portion was incubated in medium supplemented with type-II chicken collagen. The culture supernatant was collected after a 48-h incubation, and the level of the cytokines (interleukin [IL]-17, IL-6, and interferon [IFN]-γ) secreted in the culture supernatant were analyzed (Bio-Plex Pro Mouse Cytokine Th17 Panel A 6-Plex Group I, Bio-Rad Laboratories).

### Histopathological examination of knee joints

The excised knee joints were fixed in neutral-buffered 10% formalin solution. The left knee joints were decalcified in a 10% formic acid decalcification solution, and then they were cut at the femoral trochlear groove to prepare the paraffin sections. After hematoxylin and eosin (H&E) staining, the sections were histologically examined.

### Statistical analysis

The numerical data for the groups are expressed as the means ± standard deviation (SD) or standard error (SE). The arthritis scores were compared between the groups using the Steel-Dwass test. The body weight, IgG antibody titer, and secreted levels of cytokines were tested for homogeneity of distribution using Bartlett’s test. The homogeneous data were further analyzed using a one-way analysis of variance (ANOVA), and significant results were examined to determine the differences between means using Dunnett’s test. Furthermore, the heterogeneous results were analyzed using Kruskal-Wallis H-test, and the significant averages of the ranks were compared using Dunnett’s test.

A significant level was set at 5% for Bartlett test, one-way analysis, and Kruskal-Wallis H-test, and at 5% and 1% for Dunnett’s test and Steel-Dwass test.

The statistical analysis including the allocation was conducted using the JMP data analysis software (SAS Institute Japan, Co., Ltd.).

## Results

### Body weight

**Fig 1:**
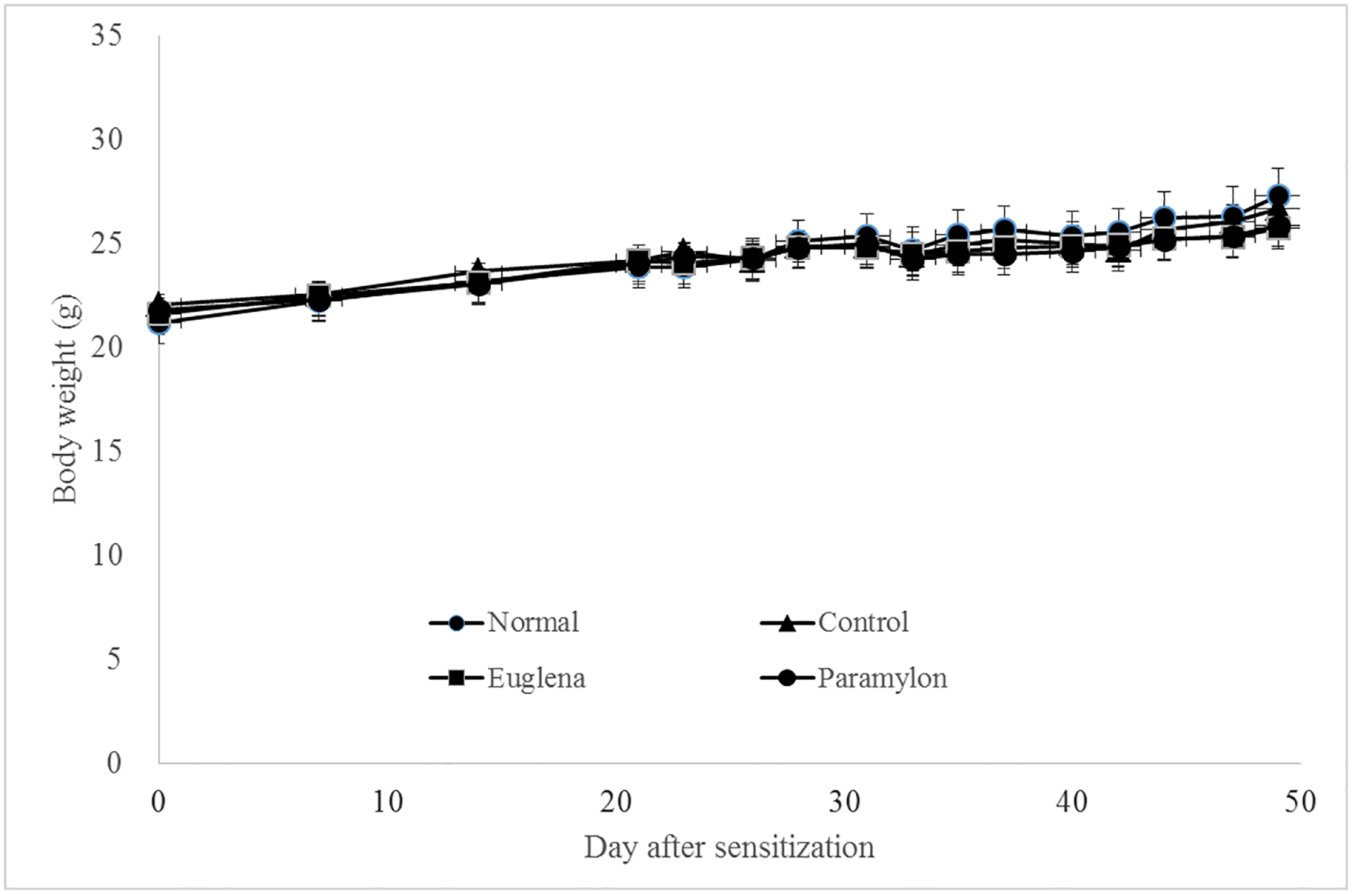
Effects of oral administration of *Euglena gracilis* Z and paramylon on body weight changes in collagen-induced arthritis mouse model. DBA/1J mice were sensitized with collagen to establish the collagen-induced arthritis mouse model. The body weight was measured once a week from the end of the quarantine period until booster immunization day, and then three times a week after the booster immunization. Each value represents the mean ± standard error (SE) of five mice.

The mean body weights of the Normal, Control, E, and P groups were 21.2 ± 0.9, 22.1 ± 0.5, 21.6 ± 0.6, and 21.8 ± 0.5, respectively. A steady and gradual increase in the body weight was observed in all groups, and no inter-group difference was detected (Fig 1). No toxic effect due to ingestion of the test substances was observed.

### Arthritis score

**Fig 2:**
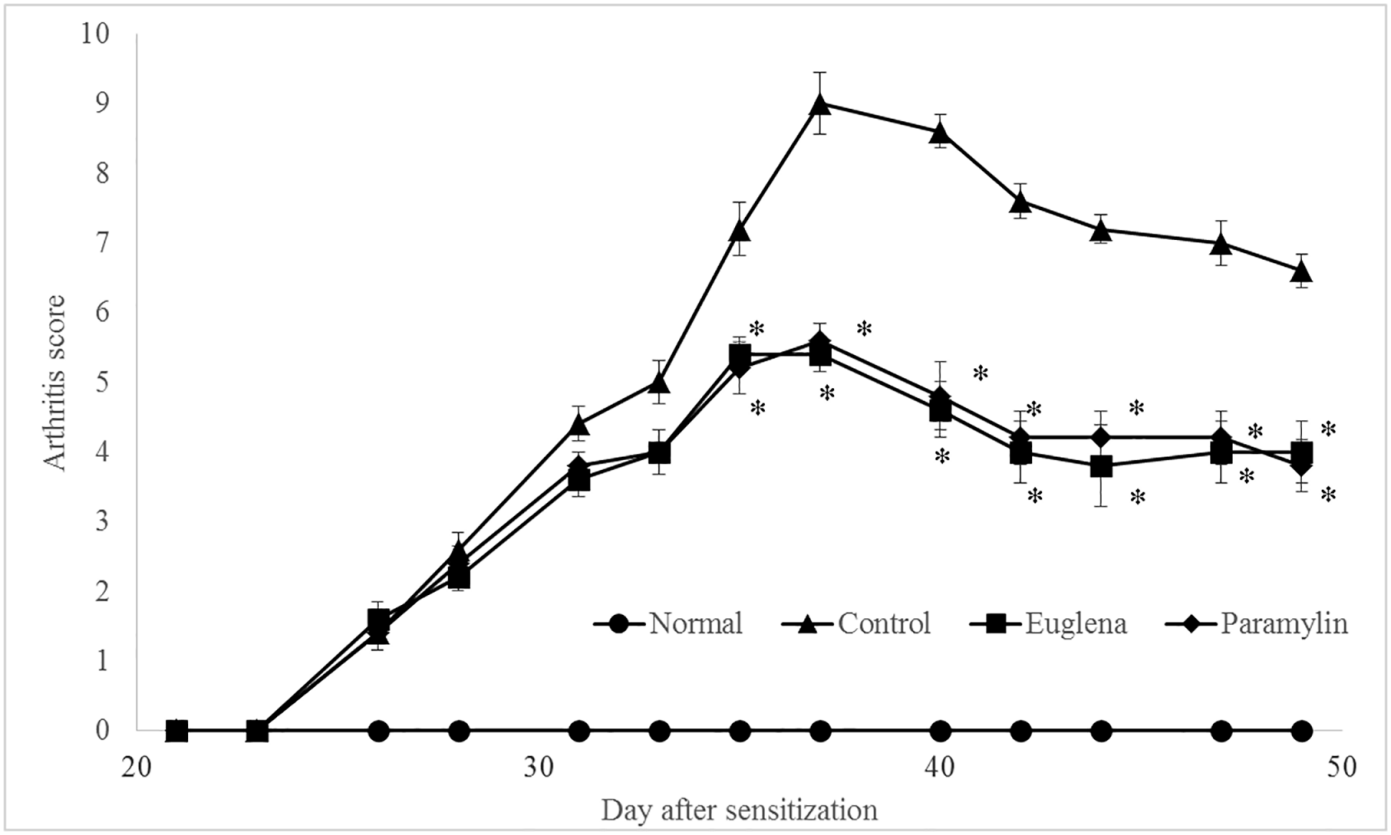
Effects of oral administration of *E*. *gracilis* Z and paramylon on arthritis score in collagen-induced arthritis mouse model. The arthritis-related condition of the extremities was evaluated three times per week. The total score of all four extremities was calculated as follows: 0: no evidence of erythema and swelling, 1: slight redness/swelling of one small joint such as phalangeal joint, 2: redness/swelling of two or more small joints, or a large joint, 3: redness/swelling of one extremity, and 4: maximum redness/swelling of the whole region of one extremity. Values are means ± standard deviation (SD) of five mice, *p < 0.05 compared to the control using Steel-Dwass test.

In the control group, the score gradually increased and reached the maximum level on 37 days (9.0 ± 0.4) after sensitization. Then, the score showed a slight decrease (Fig 2). Groups E and P showed similar gradual increases in the scores to that of the Control group; however, the scores were lower than those in Control group were, and the difference was significant. The scores of Groups E and P on day 37 day were 5.4 ± 0.2 and 5.6 ± 0.2, respectively.

### Histopathological examination

**Fig 3:**
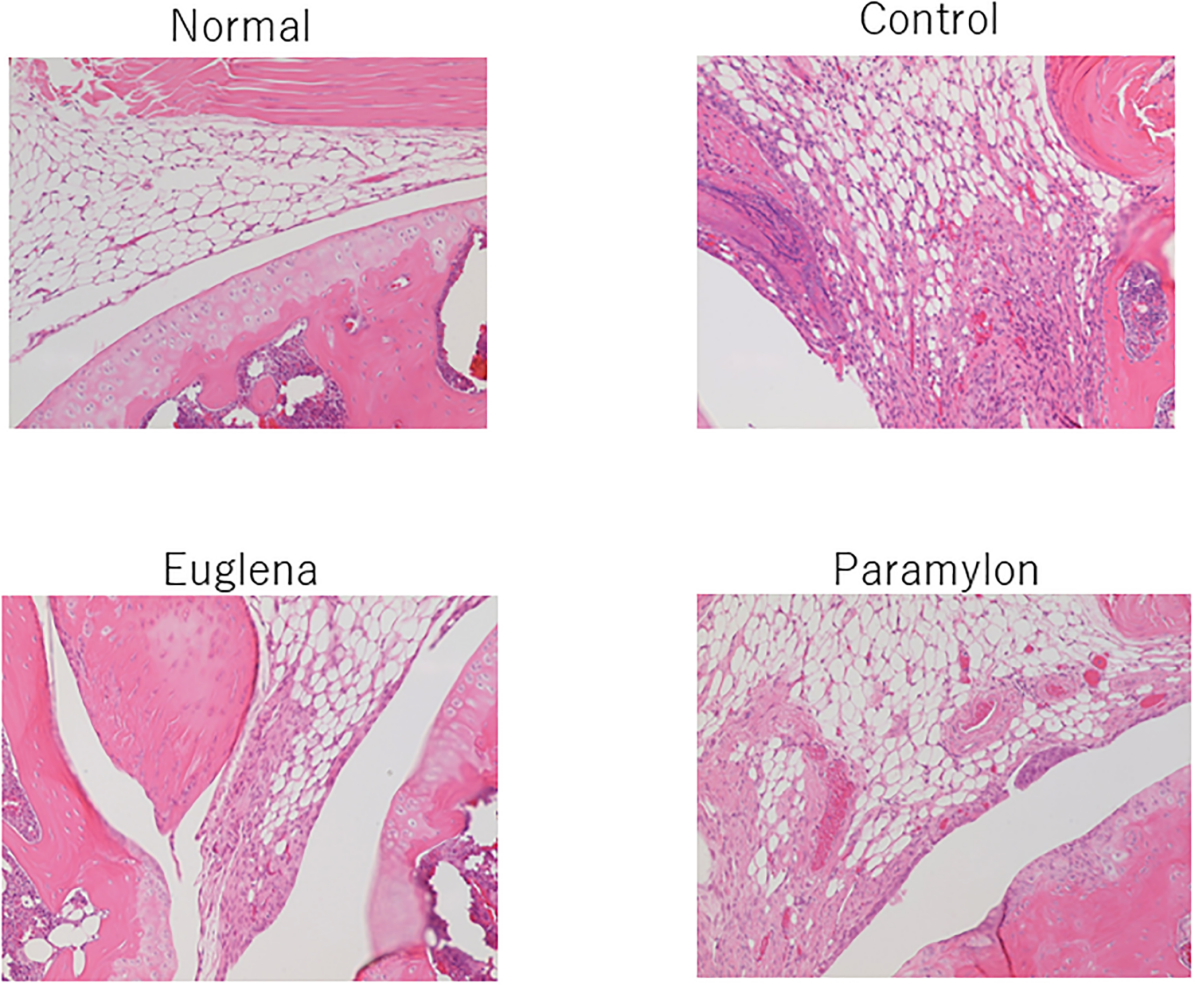
Effects of oral administration of *E*. *gracilis* Z and paramylon on histopathological changes in collagen-induced arthritis mouse model. The excised knee joints were fixed in neutral-buffered 10% formalin solution. The left knee joints were decalcified in 10% formic acid decalcification solution, and then they were cut at the femoral trochlear groove to prepare the paraffin sections. After hematoxylin and eosin (H&E) staining, the sections were histologically examined.

In the control group, slight to moderate changes were observed in the synovial membrane including edema, inflammatory cell infiltration, cell hyperplasia, granulation tissue formation, fibrosis, and exudates. In the femoral trochlear groove, mild pannus formation and destruction of articular cartilage were observed (Fig 3). In Group E, only slight granulation tissue formation was observed in the synovial membrane (Fig 3). Furthermore, Group P exhibited mild effects including inflammatory cell infiltration, granulation tissue formation, and fibrosis in the synovial membrane, and mild pannus formation and destruction of articular cartilage in the femoral trochlear groove (Fig 3).

### Serum antibody titer

**Fig 4:**
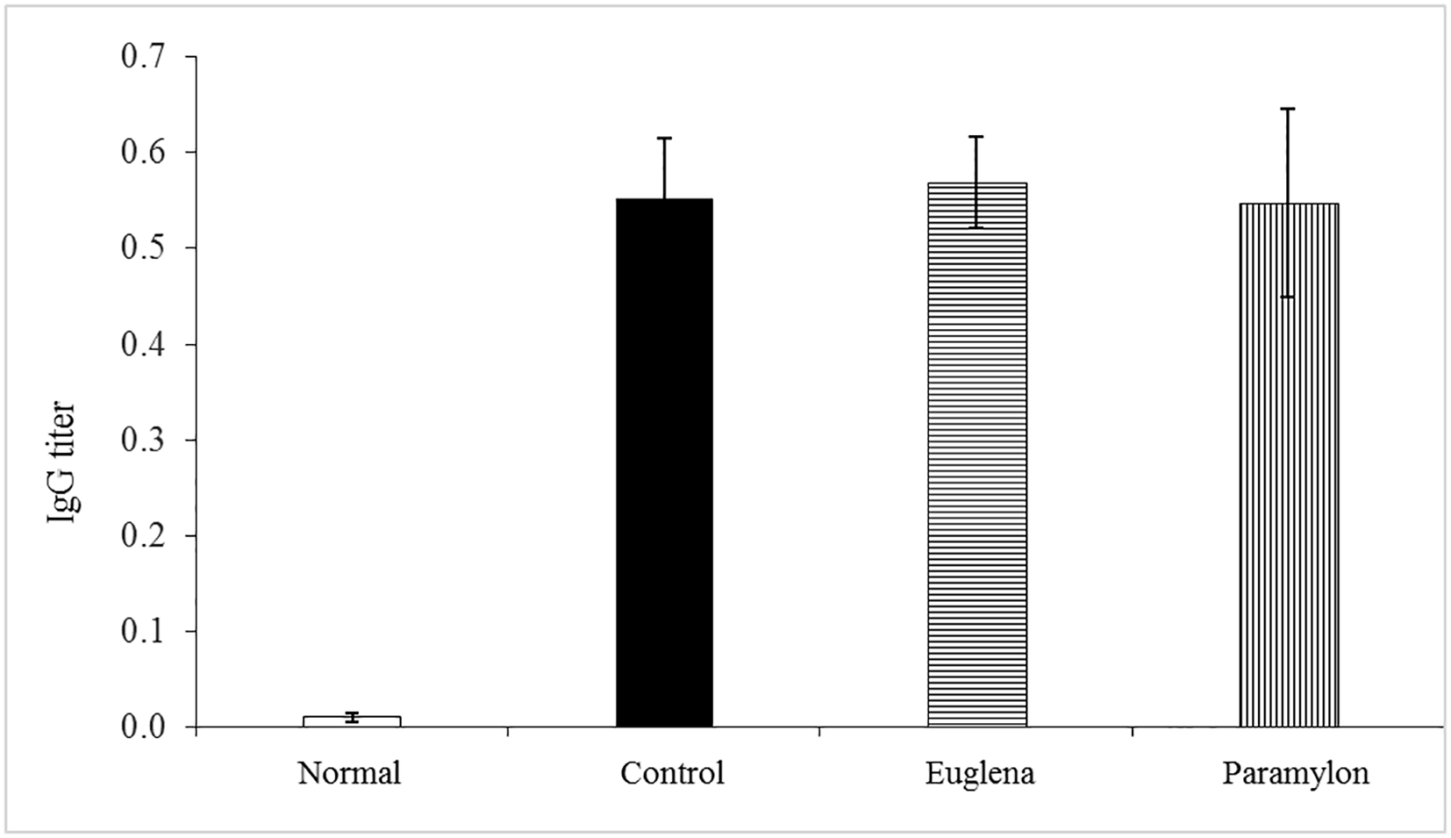
Effects of oral administration of *Euglena gracilis* Z and paramylon on serum IgG titer in collagen-induced arthritis mouse model. After terminating the test substance administration, laparotomies were performed under inhalation anesthesia with isoflurane, and blood samples were collected from the abdominal vena cava. After the blood collection, the animals were euthanized by exsanguination, and then the inguinal lymph nodes and knee joints were harvested. The serum was separated from the blood samples by centrifugation to quantify the IgG levels using an enzyme-linked immunosorbent assay (ELISA). Value are means ± standard deviation (SD) of five mice.

In the Control, E, and P groups, the IgG antibody titer against collagen was 0.552 ± 0.062, 0.568 ± 0.048, and 0.547 ± 0.098, respectively, and no difference was observed between these groups (Fig 4). These results confirmed that the sensitization with collagen established the mouse model.

### Cytokines

**Fig 5:**
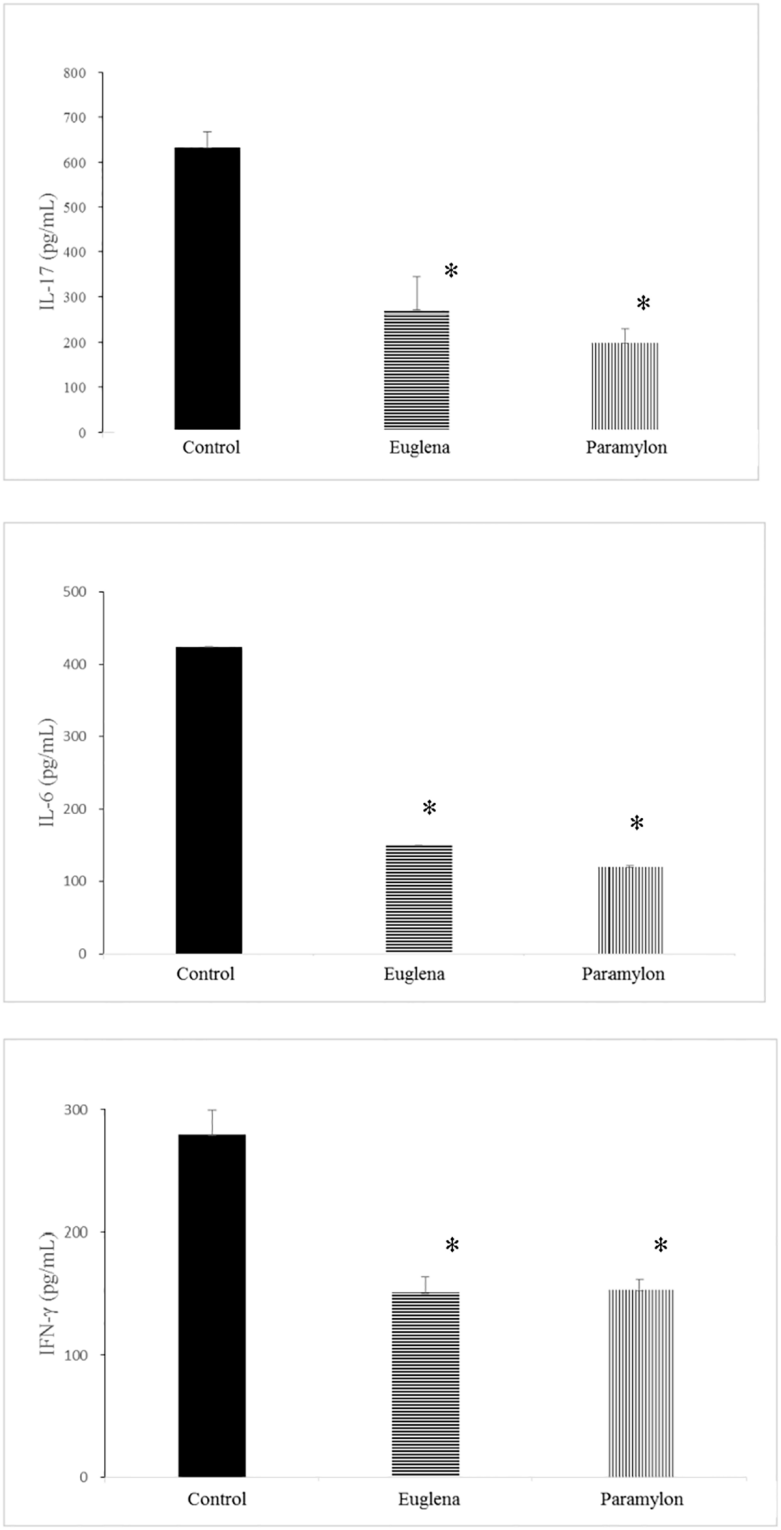
Effects of oral administration of *Euglena gracilis* Z and paramylon on cytokine production in collagen-induced arthritis mouse model. The lymphoid cells were separated from the inguinal lymph nodes and then divided into three portions, which were each incubated in medium supplemented with type-II chicken collagen. The culture supernatant was collected after a 48-h incubation, and the level of the cytokines (interleukin [IL]-17, IL-6, and interferon [IFN]-γ) secreted in the culture supernatant were analyzed (Bio-Plex Pro Mouse Cytokine Th17 Panel A 6-Plex Group I, Bio-Rad Laboratories). Values are means ± standard deviation (SD) of five mice. *p < 0.05 compared to the control using Dunnett’s test.

The levels of IL-17, IL-6, and IFN-γ cytokines in the Control group were 633.72 ± 35.39, 424.01 ± 43.43, and 279.62 ± 20.02 pg/mL, respectively. In Groups E and P, significantly lower levels of the IL-17, IL-6, and IFN-γ cytokines than those in the Control group were observed (Fig 5).

## Discussion

The symptom-relieving effects of *E*. *gracilis* Z and its carbohydrate storage substance, paramylon, on rheumatoid arthritis were evaluated in a collagen-induced arthritis mouse model. *E*. *gracilis* Z and paramylon were administered for 24 days as a 2% dietary supplement after confirmation of arthritis symptoms. During the administration period, signs of arthritis were observed and scored visually. After terminating the test substance administration, blood samples were collected, and the knee joints were excised. Then, the serum IgG concentration and levels of cytokines (IL-17, IL-6, and IFN-γ) secreted into the culture supernatant from the extracted lymph nodes, were determined and the knee joints were histopathologically examined.

In the control group, a daily increase in the arthritis score as well as histopathological changes was observed. In contrast, the *E*. *gracilis* Z and paramylon groups showed lower transition rates of arthritis scores compared to the control group with statistical significance.

The onset of arthritis involves many immune cells, and anti-TNF-α and anti-IL-1 antibodies are used as treatment options [15,16]. The collagen-induced arthritis mouse model is known as a human rheumatoid arthritis model and is reported to exhibit mRNA increases in TNF-α, IL-1β, IL-6, and IL-18 during the progression of arthritis [17]. Rheumatoid arthritis was initially considered a Th1-type autoimmune disease. However, deterioration of arthritis was reported in IFN-γ receptor knockout mice [18–20]. Similar pathological deterioration was reported in a mouse model of the autoimmune disease multiple sclerosis and experimental autoimmune encephalomyelitis (EAE) administered an anti-IFN-γ antibody and in IFN-γ knockout mice [21–25].

Based on these results, autoimmune diseases such as rheumatoid arthritis and multiple sclerosis are currently considered Th17- and not Th1-type pathologies [26]. Specifically, in rheumatoid arthritis, naïve helper T-cells are considered to differentiate into Th17 cells by the action of molecules such as transforming growth factor (TGF)-β, IL-6, and IL-23. Subsequently, Th17 cells produce factors such as IL-17 and receptor activator of nuclear factor kappa-B ligand (RANKL), which induce inflammation of the synovial membrane and osteoclast leading to progression of the pathology [26]. In contrast, IFN-γ is known to suppress cell differentiation into Th17 cells by promoting the differentiation of naïve T-cell to Th1 cell. This explains the deterioration associated with collagen-induced arthritis in IFN-γ knockout mice since differentiation of naïve T-cell to Th17 cell is not suppressed in IFN-γ knockout mouse.

*E*. *gracilis* Z contains paramylon, a carbohydrate storage substance. Paramylon is a polysaccharide with a β-1,3-glucan structure. The sample used in the present study contained paramylon at a concentration of approximately 30%, and the function of paramylon is promising. Furthermore, β-1, 3-glucan is a polysaccharide that is widely distributed in nature including in fungi, bacteria, and plants, and it exhibits several physiological activities in the human body. Among these activities, immune regulation is the most reported [27,28]. The immunostimulatory effect of β-glucans is mediated via the innate immune system and defends against pathogens [29]. Following ingestion, β-glucans affect the mucosal immune system in the gastrointestinal tract. The uptake of microorganisms from the intestinal lumen is undertaken by the M cells of Peyer’s patches (PPs) in the small intestine. Subsequently, these cells present the antigen, at their basal surfaces, to the immune cells such as macrophages and dendritic cells [30].

To identify the pathogens, the receptors specific to their cell walls are enriched on the surface of immune cells. Several pattern recognition receptors target β-glucans present in the cell walls of microorganisms. This explains why β-glucans can stimulate the intestinal mucosal immune system. Although it is not clear how β-glucans penetrate the epithelial cell lining, some epithelial cells such as M cells may actively incorporate β-glucans (similar to other antigens).

Dectin-1, which is expressed on the surfaces of macrophages and dendritic cells, is reported to act as the primary receptor for β-glucans, which subsequently display immunostimulatory effects [31,32]. β-glucans recognized by Dectin-1 promote the intracellular uptake of β-glucan granules [33] and the secretion of proinflammatory cytokines [34–36] by activating a tyrosine kinase Syk, and the transcription factor, nuclear factor-κB, which is responsible for multiple immune reactions [37–39].

The effects of paramylon are similar to those of other β-glucans. This suggests that paramylon also functions by being transported across the epithelial cells, is recognized by macrophages via the Dectin-1 receptor, and induces the Th1 cell response. The size of paramylon is typically 2–3 μm, which is similar to the size of pathogenic bacteria. This suggests that paramylon is potentially transported across the epithelial cells using the same mechanism of pathogenic bacteria. The crystalline structure of paramylon consists of triple helices [40,41], which are recognized by the β-glucan receptor Dectin-1, implying that paramylon presumably activates Dectin-1.

These observations indicate that paramylon would be expected to exhibit some effects on the immune system. In the present study, *E*. *gracilis* Z and paramylon suppressed the onset of arthritis and decreased the secretion of cytokines including IL-17 and IL-6. These results suggest that *E*. *gracilis* Z and its carbohydrate storage substance suppressed the differentiation to Th17 cell and subsequently suppressed inflammation of synovial membrane and destruction of the cartilage. In addition, 7 weeks after collagen sensitization, the level of the ratio of Th17 and Treg cells in CD4-positive T cells was analyzed using a flowcytometer. The result showed that the ratio of Th17 cells in CD4-positive T cells of group E was equal to that of the Control group. Furthermore, the ratio of Th17 cells to CD4-positive T cells of group P was lower than that of the Control group. This result indicates that *E*. *gracilis* and paramylon suppressed differentiation of cells into Th17 cells. In autoimmune diseases, granulocyte-macrophage colony-stimulating factor (GM-CSF) has been reported to promote secretion, induce Treg cells, and ameliorate symptoms such as those of rheumatoid arthritis, Crohn’s disease [42], and myasthenia gravis [43–45]. In addition, it is also clear that autoimmune diabetes (type I diabetes) requires IL-2 for the stable maintenance of Treg cells [46]. Furthermore, it has been reported that sensitization of collagen on the anterior chamber (AC) of the eyes induces Treg cells to show immune deviations and alleviates symptoms such as rheumatoid arthritis [47,48]. We also examined the possibility that *Euglena* and paramylon induce Treg cell, but no significant difference was found in Foxp3 positive rate compared to the control group (S1 Fig). Therefore, it appears that other factors may mediate the rheumatic symptom-relieving effects of *E*. *gracilis* and paramylon. Moreover, suppression IFN-γ secretion was noted. Paramylon has been shown to improve the symptoms of atopic dermatitis and decrease the secretion of IL-4, IFN-γ, IL-18, and IL-12 in NC/Nga mice [10]. Based on these results, it is postulated that paramylon regulates excessive immune responses, including the activities of Th1 and Th2 [10].

The decrease in the IFN-γ level observed in the present study is in agreement with these results. Therefore, it has been suggested that *E*. *gracilis* Z and its carbohydrate storage substance regulate Th17 as well as Th1 and Th2, and have a regulatory function on helper T-cell. The mechanisms of action of *E*. *gracilis* Z have not been identified. However, since *E*. *gracilis* Z and paramylon exhibited comparable symptom-relieving activity, paramylon might be the active constituent of *E*. *gracilis* Z.

The results of this study indicate that *E*. *gracilis* Z and paramylon suppressed the onset of arthritis in the collagen-induced arthritis mouse model. Especially, its suppressive effect on the IL-17 Th17 type cytokine was observed and considered to be mediated by their regulatory effects on Th1- related immune responses.

## Supporting information

S1 Fig Analysis of Th17 and Treg phenotype. The lymphoid cells were separated from the inguinal lymph nodes. The phenotype was characterized by staining for phycoerythrin (PE)-conjugated anti-IL-17A and allophycocyanin (APC)-conjugated anti-Foxp3.
(TIF)

S1 Table Body weight. DBA/1J mice were sensitized with collagen to establish the collagen-induced arthritis mouse model. The body weight was measured once a week from the end of the quarantine period until booster immunization day, and then three times a week after the booster immunization.
(DOCX)

S2 Table Arthritis score. The arthritis-related condition of the extremities were evaluated three times per week (Monday, Wednesday, and Friday). The total score of all four extremities was calculated as follows: 0: no evidence of erythema and swelling, 1: slight redness/swelling of one small joint such as phalangeal joint, 2: redness/swelling of two or more small joints, or a large joint, 3: redness/swelling of one extremity, and 4: maximum redness/swelling of the whole region of one extremity.
(DOCX)

S3 Table Serum IgG titer. After terminating test substance administration, laparotomies were performed under inhalation anesthesia with isoflurane, and blood samples were collected from the abdominal vena cava. After the blood collection, the animals were euthanized by exsanguination, and then the inguinal lymph nodes and knee joints were harvested. The serum was separated from the blood samples by centrifugation to quantify the IgG levels using an enzyme-linked immunosorbent assay (ELISA).
(DOCX)

S4 Table Cytokine production. The lymphoid cells were separated from the inguinal lymph nodes and then divided into three portions, which were each incubated in medium supplemented with type-II chicken collagen. The culture supernatant was collected after a 48-h incubation, and the levels of cytokines (interleukin [IL]-17, IL-6, and interferon [IFN]-γ) secreted in the culture supernatant were analyzed (Bio-Plex Pro Mouse Cytokine Th17 Panel A 6-Plex Group I, Bio-Rad Laboratories).
(DOCX)

S5 Table Analysis of Th17 and Treg phenotype. The lymphoid cells were separated from the inguinal lymph nodes. The phenotype was characterized by staining for phycoerythrin (PE)-conjugated anti-IL-17A and allophycocyanin (APC)-conjugated anti-Foxp3. Not significantly different from control using Dunnett’s test.
(DOCX)

## References

[1] Rossini M, Caimmi C, Bernardi D, Rossi E, Viapiana O, De Rosa M. Epidemiology and hospitalization rate of rheumatoid arthritis patients in real world setting in Italy. Ann Rheum Dis. 2013;72: 409.

[2] Widdifield J, Paterson JM, Bernatsky S, Tu K, Thorne JC, Bombardier C. Epidemiology of rheumatoid arthritis in a universal public health care system: results from the Ontario RA Administrative Database (Orad). Ann Rheum Dis. 2013;72: 549–550.

[3] Seki N, Sudo Y, Yoshida T, Sugihara S, Fujita T, Sakuma S, et al Type II collagen-induced murine arthritis. I. Induction and perpetuation of arthritis require synergy between humoral and cell-mediated immunity. J Immunol. 1998;140: 1477–1484. 3257978

[4] Ricard-Blum S, Ruggiero F. The collagen superfamily: from the extracellular matrix to the cell membrane. Pathol Biol. 2005;53: 430–442. doi: 10.1016/j.patbio.2004.12.024 16085121 10.1016/j.patbio.2004.12.024

[5] Trentham DE, Townes AS, Kang AH. Autoimmunity of type II collagen an experimental model of arthritis. J Exp Med. 1977;146: 857–868. 894190 10.1084/jem.146.3.857 PMC2180804

[6] Courtenay JS, Dallman MJ, Dayan AD, Martin A, Mosada B. Immunization against heterologous type II collagen-induced arthritis in mice. Nature. 1980;283: 666–668. 6153460 10.1038/283666a0

[7] Watanabe T, Shimada R, Matsuyama A, Yuasa M, Sawamura H, Yoshida E, et al Antitumor activity of the β-glucan paramylon from *Euglena* against preneoplastic colonic aberrant crypt foci in mice. Food Funct. 2013;4; 1685–1690. doi: 10.1039/c3fo60256g 24104447 10.1039/c3fo60256g

[8] Shimada R, Fujita M, Yuasa M, Sawamura H, Watanabe T, Nakashima A, et al Oral administration of green algae, *Euglena gracilis*, inhibits hyperglycemia in OLETF rats, a model of spontaneous type 2 diabetes. Food Funct. 2016;7: 4655–4659 doi: 10.1039/c6fo00606j 27775129 10.1039/c6fo00606j

[9] Kondo Y, Kato A, Hojo H, Nozoe S, Takeuchi M, Ochi K. Cytokine-related immunopotentiating activities of Paramylon, a β-(1→3) glucan from *Euglena gracilis* Z. J Pharmacobio Dyn. 1992;15: 617–621 1289496 10.1248/bpb1978.15.617

[10] Sugiyama A, Hata S, Suzuki K, Yoshida E, Nakano R, Mitra S, et al Oral administration of Paramylon, a β-1,3-D-glucan isolated from *Euglena gracilis* Z inhibits development of atopic dermatitis-like skin lesions in NC/Nga mice. J Vet Med Sci. 2010;72: 755–763. 20160419 10.1292/jvms.09-0526

[11] Sugiyama A, Suzuki K, Mitara S, Arashida R, Yoshida E, Nakano R, et al Hepatoprotective effects of paramylon, a β-1,3-D-Glucan isolated from *Euglena gracilis* Z on acute liver injury induced by carbon tetrachloride in rat. J Vet Med Sci. 2009;71: 885–890. 19652474 10.1292/jvms.71.885

[12] Koizumi N, Sakagami H, Utsumi A, Fujinaga S, Takeda M, Asano K, et al Anti-HIV (human immunodeficiency virus) activity of sulfated paramylon. Antiviral Res. 1993; 21: 1–14. 8317920 10.1016/0166-3542(93)90063-o

[13] Sakagami H, Kikuchi K, Takeda M, Sato T, Ichikawa S, Fujimaki M, et al Macrophage stimulation activity of antimicrobial N. N-dimethylamino ethyl paramylon. In Vivo. 1991;5: 101–105 1768776

[14] Bernadeta N, Marta CL, Malgorzata S, Andrzej G, Ewa K, Sapria GF, et al *Lactobacillus rhamnosus* Exopolysaccharide ameliorates arthritis induced by the systemic injection of collagen and lipopolysaccharide in DBA/1 mice. Arch Immunol Ther Exp. 2012; 60: 211–220 10.1007/s00005-012-0170-5 22484803

[15] Criscione LG, Stclair EW. Tumor necrosis factor-alpha antagonists for the treatment of rheumatoid disease. Curr Opin Pharmacol. 2002;14: 204–211 10.1097/00002281-200205000-00002 11981314

[16] Taylor PC. Anti-cytokines and cytokines in the treatment of rheumatoid, arthritis. Curr Pharm Res. 2003;9: 1091–1106 10.2174/1381612033454991 12769749

[17] Shinobe R, Sato M, Takemura M, Shimizu K, Koishi H, Tanaka R, et al Cytokine profiles in mice with arthritis induced by anti-type II collagen monoclonal antibody plus lipopolysaccharide. Jpn J Clin Chem. 2008;37: 53–62

[18] Manoury-Schwartz B, Chiocchia G, Bessis N, Abehsira-Amar O, Battenx F, Muller S, et al High susceptibility to collagen-induced arthritis in mice lacking IFN-gamma receptors. J Immunol. 1997; 158: 5501–5506 9164973

[19] Vemeire K, Heremans H, Vandeputte M, Huang S, Billiar A, Matthys P. Accelerated collagen-induced arthritis in IFN-gamma receptor-deficient mice. J Immunol. 1997; 158: 5507–5513 9164974

[20] Chu CQ, Song Z, Mayton L, Wu B, Wooley PH. IFN gamma deficient C57BL/6 (H-2b) mice develop collagen induced arthritis with predominant usage of T cell receptor Vbeta6 and Vbeta8 in arthritic joints. Ann Rheum Dis. 2003;62: 983–990 doi: 10.1136/ard.62.10.983 12972478 10.1136/ard.62.10.983 PMC1754310

[21] Voorthuis JA, Uitdehaag BM, De Groot CJ, Goede PH, van der Meide PH, Dijkstra CD. Suppression of experimental allergic encephalomyelitis by intraventricular administration of interferon-gamma in Lewis rats. Clin Exp Immunol. 1990;81: 183–188 2117508 10.1111/j.1365-2249.1990.tb03315.x PMC1535058

[22] Willenborg DO, Fordham S, Bernard CC, Cowden WB, Ramshaw IA. IFN-gamma plays a critical down-regulatory role in the induction and effector phase of myelin oligodendrocyte glycoprotein-induced autoimmune encephalomyelitis. J Immunol. 1996;157: 3223–3227 8871615

[23] Krakowski M, Owens T. Interferon-gamma confers resistance to experimental allergic encephalomyelitis. Eur J Immunol. 1996;26: 1641–1646 doi: 10.1002/eji.1830260735 8766573 10.1002/eji.1830260735

[24] Billiau A, Heremans H, Vandekerckhove F, Gijkmans R, Sobis H, Manlepas E, et al Enhancement of experimental allergic encephalomyelitis in mice by antibodies against IFN-gamma. J Immunol. 1988;140: 1506–1510 3126227

[25] Ferber IA, Brocke S, Tayler-Edwards C, Ridgway W, Dinisco C, Steinman L, et al Mice with a disrupted IFN-gamma gene are susceptible to the induction of experimental autoimmune encephalomyelitis (EAE). J Immunol. 1996;156: 5–7 8598493

[26] Sato K. Th17 cells and rheumatoid arthritis—From the standpoint of osteoclast differentiation–. Allergol Int. 2008;27: 109–114 10.2332/allergolint.R-07-158 18427163

[27] Taguchi T, Furue H, Kimura T, Kondo T, Hattori T, Ogawa N. Clinical efficacy of lentinan on neoplastic diseases. Adv Exp Med Biol. 1983;166: 181–187. 6650280 10.1007/978-1-4757-1410-4_15

[28] Chen J, Zhang XD, Jiang Z. The application of fungal β-glucans for the treatment of colon cancer. Anticancer Agents Med Chem. 2013;13: 725–730. 23293888 10.2174/1871520611313050007

[29] Macpherson AJ, Harris NL. Interactions between commensal intestinal bacteria and the immune system. Nat Rev Immunol. 2004;4: 478–485. doi: 10.1038/nri1373 15173836 10.1038/nri1373

[30] Kraehenbuhl JP, Neutra MR. Epithelial M cells: differentiation and function. Annu Rev Cell Dev Biol. 2000;16: 301–332. doi: 10.1146/annurev.cellbio.16.1.301 11031239 10.1146/annurev.cellbio.16.1.301

[31] Brown GD, Gordon S. Immune recognition. A new receptor for beta-glucans. Nature. 2001;13: 36–37. 10.1038/35092620 11544516

[32] Ariizumi K, Shen GL, Shikano S, Xu S, Ritter R, Kumamoto T, et al Identification of a novel, dendritic cell-associated molecule, dectin-1, by subtractive cDNA cloning. J Biol Chem. 2000;275: 20157–20167. doi: 10.1074/jbc.M909512199 10779524 10.1074/jbc.M909512199

[33] Mansour MK, Tam JM, Khan NS, Seward M, Davids PJ, Puranam S, et al Dectin-1 activation controls maturation of β-1,3-glucan-containing phagosomes. J Biol Chem 2013; 288:16043–16054. doi: 10.1074/jbc.M113.473223 23609446 10.1074/jbc.M113.473223 PMC3668760

[34] Kankkunen P, Teirila L, Rintahaka J, Alenius H, Wolff H, Matikainen S. (1,3)-Beta-glucans activate both dectin-1 and NLRP3 inflammasome in human macrophages. J Immunol. 2010;184: 6335–63342. doi: 10.4049/jimmunol.0903019 20421639 10.4049/jimmunol.0903019

[35] Masuda Y, Inoue H, Ohta H, Miyake A, Konishi M, Nanba H. Oral administration of soluble β-glucans extracted from *Grifola frondosa* induces systemic antitumor immune response and decreases immunosuppression in tumor-bearing mice. Int J Cancer. 2013;133: 108–119. doi: 10.1002/ijc.27999 23280601 10.1002/ijc.27999

[36] Masuda Y, Togo T, Mizuno S, Konishi M, Nanba H. Soluble β-glucan from *Grifola frondosa* induces proliferation and Dectin-1/Syk signaling in resident macrophages via the GM-CSF autocrine pathway. J Leukoc Biol. 2012;91: 547–556. doi: 10.1189/jlb.0711386 22028332 10.1189/jlb.0711386

[37] Tada R, Ikeda F, Aoki K, Yoshikawa M, Kato Y, Adachi Y, et al Barley-derived beta-D-glucan induces immunostimulation via a dectin-1-mediated pathway. Immunol Lett. 2009;123: 144–148. doi: 10.1016/j.imlet.2009.03.005 19428562 10.1016/j.imlet.2009.03.005

[38] Adachi Y. Role of the 1, 3-β-D-glucan receptor dectin-1 in fungal infection and activation of innate and anti-tumor immunity. Trends Glycosci Glycotechnol. 2007;19: 195–207.

[39] Reid DM, Gow NAR, Brown GD. Pattern recognition: recent insights from Dectin-1. Curr Opin Immunol. 2009;21: 30–37. doi: 10.1016/j.coi.2009.01.003 19223162 10.1016/j.coi.2009.01.003 PMC2684021

[40] Kiss JZ, Roberts EM, Brown RM, Triemer RE. X-ray and dissolution studies of paramylon storage granules from *Euglena*. Protoplasma. 1988;146: 150–156.

[41] Chuah CT, Sarko A, Deslandes Y, Marchessault RH. Packing analysis of carbohydrates and polysaccharides. Part 14. Triple-helical crystalline structure of curdlan and paramylon hydrates. Macromolecules. 1983;16: 1375–1382.

[42] Gathungu G, Kim MO, Ferguson JP, Sharma Y, Zhang W, Ng SM, et al Granulocyte-Macrophage colony-stimulating factor auto-antibodies: A marker of aggressive Crohn’s disease. Inflamm Bowel Dis. 2013;19: 1671–180. doi: 10.1097/MIB.0b013e318281f506 23749272 10.1097/MIB.0b013e318281f506 PMC3707315

[43] Rowin J, Thiruppathi M, Arhebamen E, Sheng J, Prabhakar BS, Meriggioli MN. Granulocyte-macrophage colony-stimulating factor treatment of a patient in myasthenic crisis: Effects on regulatory T cells. Muscle Nerve. 2012;46: 449–453. doi: 10.1002/mus.23488 22907239 10.1002/mus.23488 PMC3428740

[44] Bhattacharya P, Budnick I, Singh M, Thiruppathi M, Alharshawi K, Elshabrawy H, et al Dual role of GM-CSF as a pro-inflammatory and a regulatory cytokine: implications for immune therapy. J Interferon Cytokine Res. 2015;35: 585–599. doi: 10.1089/jir.2014.0149 25803788 10.1089/jir.2014.0149 PMC4529096

[45] Bhattacharya P, Thiruppathi M, Elshabrawy HA, Alharshawi K, Kumar P, Prabhakar BS. GM-CSF: An immune modulatory cytokine that can suppress autoimmunity. Cytokine. 2015;75: 261–271. doi: 10.1016/j.cyto.2015.05.030 26113402 10.1016/j.cyto.2015.05.030 PMC4553090

[46] Haddad CS, Bhattacharya P, Alharshawi K, Marinelarena A, Kumar P, El-Sayed O, et al Age-dependent divergent effects of OX40L treatment on the development of diabetes in NOD mice. Autoimmunity. 2016;49: 298–311. doi: 10.1080/08916934.2016.1183657 27245356 10.1080/08916934.2016.1183657 PMC5042830

[47] Farooq SM, Ashour HM. Type II collagen induces peripheral tolerance in BALB/c mice via the generation of CD8+ T regulatory cells. PLoS One. 2012;7: e48635 doi: 10.1371/journal.pone.0048635 23133648 10.1371/journal.pone.0048635 PMC3487721

[48] Farooq SM, Kumar A, Ashour HM. Eye-mediated immune tolerance to Type II collagen in arthritis-prone strains of mice. J Cell Mol Med. 2014;18: 2512–2518 doi: 10.1111/jcmm.12376 25211510 10.1111/jcmm.12376 PMC4302655

