# Supplementary figures and images for: Euglena gracilis Z and its carbohydrate storage substance relieve arthritis symptoms by modulating Th17 immunity
Source: PLoS One. 2018 Feb 1;13(2):e0191462. doi: 10.1371/journal.pone.0191462 (PMC5794092; doi:10.1371/journal.pone.0191462)

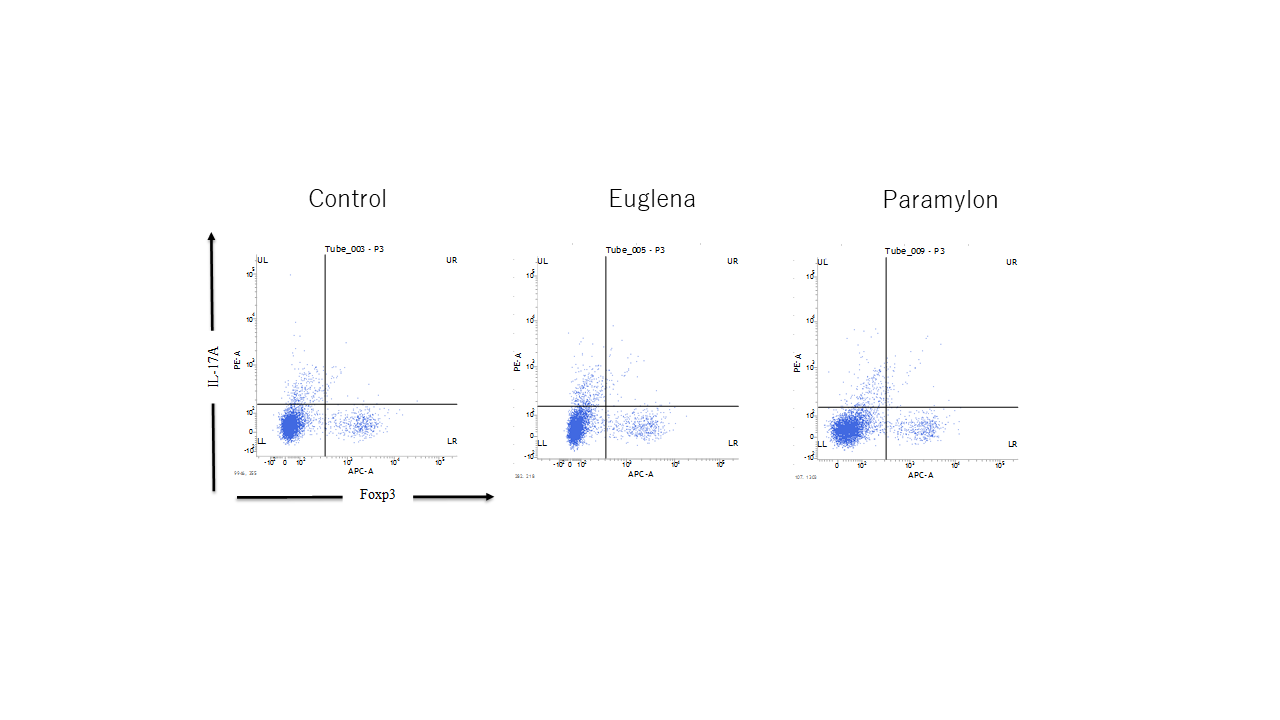

Supplement: S1 Fig — The lymphoid cells were separated from the inguinal lymph nodes. The phenotype was characterized by staining for phycoerythrin (PE)-conjugated anti-IL-17A and allophycocyanin (APC)-conjugated anti-Foxp3. (TIF) [file pone.0191462.s001.TIF]
