# Supplementary material for: Euglena gracilis Z and its carbohydrate storage substance relieve arthritis symptoms by modulating Th17 immunity
Source: PLoS One. 2018 Feb 1;13(2):e0191462. doi: 10.1371/journal.pone.0191462 (PMC5794092; doi:10.1371/journal.pone.0191462)
Supplement: S1 Table — DBA/1J mice were sensitized with collagen to establish the collagen-induced arthritis mouse model. The body weight was measured once a week from the end of the quarantine period until booster immunization day, and then three times a week after the booster immunization. (DOCX) [file pone.0191462.s002.docx]

**S1 Table. Body weight**

DBA/1J mice were sensitized with collagen to establish the collagen-induced arthritis mouse model. The body weight was measured once a week from the end of the quarantine period until booster immunization day, and then three times a week after the booster immunization.
