# Supplementary material for: Euglena gracilis Z and its carbohydrate storage substance relieve arthritis symptoms by modulating Th17 immunity
Source: PLoS One. 2018 Feb 1;13(2):e0191462. doi: 10.1371/journal.pone.0191462 (PMC5794092; doi:10.1371/journal.pone.0191462)
Supplement: S2 Table — The arthritis-related condition of the extremities were evaluated three times per week (Monday, Wednesday, and Friday). The total score of all four extremities was calculated as follows: 0: no evidence of erythema and swelling, 1: slight redness/swelling of one small joint such as phalangeal joint, 2: redness/swelling of two or more small joints, or a large joint, 3: redness/swelling of one extremity, and 4: maximum redness/swelling of the whole region of one extremity. (DOCX) [file pone.0191462.s003.docx]

**S2 Table. Arthritis score**

The arthritis-related condition of the extremities were evaluated three times per week (Monday, Wednesday, and Friday). The total score of all four extremities was calculated as follows: 0: no evidence of erythema and swelling, 1: slight redness/swelling of one small joint such as phalangeal joint, 2: redness/swelling of two or more small joints, or a large joint, 3: redness/swelling of one extremity, and 4: maximum redness/swelling of the whole region of one extremity.
