# Supplementary material for: Euglena gracilis Z and its carbohydrate storage substance relieve arthritis symptoms by modulating Th17 immunity
Source: PLoS One. 2018 Feb 1;13(2):e0191462. doi: 10.1371/journal.pone.0191462 (PMC5794092; doi:10.1371/journal.pone.0191462)
Supplement: S3 Table — After terminating test substance administration, laparotomies were performed under inhalation anesthesia with isoflurane, and blood samples were collected from the abdominal vena cava. After the blood collection, the animals were euthanized by exsanguination, and then the inguinal lymph nodes and knee joints were harvested. The serum was separated from the blood samples by centrifugation to quantify the IgG levels using an enzyme-linked immunosorbent assay (ELISA). (DOCX) [file pone.0191462.s004.docx]

**S3 Table. Serum IgG titer**

After terminating test substance administration, laparotomies were performed under inhalation anesthesia with isoflurane, and blood samples were collected from the abdominal vena cava. After the blood collection, the animals were euthanized by exsanguination, and then the inguinal lymph nodes and knee joints were harvested. The serum was separated from the blood samples by centrifugation to quantify the IgG levels using an enzyme-linked immunosorbent assay (ELISA).
