# Supplementary material for: Euglena gracilis Z and its carbohydrate storage substance relieve arthritis symptoms by modulating Th17 immunity
Source: PLoS One. 2018 Feb 1;13(2):e0191462. doi: 10.1371/journal.pone.0191462 (PMC5794092; doi:10.1371/journal.pone.0191462)
Supplement: S4 Table — The lymphoid cells were separated from the inguinal lymph nodes and then divided into three portions, which were each incubated in medium supplemented with type-II chicken collagen. The culture supernatant was collected after a 48-h incubation, and the levels of cytokines (interleukin [IL]-17, IL-6, and interferon [IFN]-γ) secreted in the culture supernatant were analyzed (Bio-Plex Pro Mouse Cytokine Th17 Panel A 6-Plex Group I, Bio-Rad Laboratories). (DOCX) [file pone.0191462.s005.docx]

**S4 Table. Cytokine production**

The lymphoid cells were separated from the inguinal lymph nodes and then divided into three portions, which were each incubated in medium supplemented with type-II chicken collagen. The culture supernatant was collected after a 48-h incubation, and the levels of cytokines (interleukin [IL]-17, IL-6, and interferon [IFN]-γ) secreted in the culture supernatant were analyzed (Bio-Plex Pro Mouse Cytokine Th17 Panel A 6-Plex Group I, Bio-Rad Laboratories).
