# Supplementary material for: Euglena gracilis Z and its carbohydrate storage substance relieve arthritis symptoms by modulating Th17 immunity
Source: PLoS One. 2018 Feb 1;13(2):e0191462. doi: 10.1371/journal.pone.0191462 (PMC5794092; doi:10.1371/journal.pone.0191462)
Supplement: S5 Table — The lymphoid cells were separated from the inguinal lymph nodes. The phenotype was characterized by staining for phycoerythrin (PE)-conjugated anti-IL-17A and allophycocyanin (APC)-conjugated anti-Foxp3. Not significantly different from control using Dunnett’s test. (DOCX) [file pone.0191462.s006.docx]

**S5 Table. Analysis of Th17 and Treg phenotype.**

The lymphoid cells were separated from the inguinal lymph nodes. The phenotype was characterized by staining for phycoerythrin (PE)-conjugated anti-IL-17A and allophycocyanin (APC)-conjugated anti-Foxp3. Not significantly different from control using Dunnett’s test.
